# Design and evaluation of a mobile application for enhancing farm management and performance assessment in fattening beef cattle

**DOI:** 10.5455/javar.2024.k766

**Published:** 2024-03-31

**Authors:** Wiranut Thannithi, Payungsuk Intawicha, Phuwitsorn Phuwisaranakom, Sureeporn Saengwong

**Affiliations:** 1 Division of Animal Science, School of Agriculture and Natural Resources, University of Phayao, Phayao, Thailand; 2 Department of Computer Engineering, School of Information and Communication Technology, University of Phayao, Phayao, Thailand

**Keywords:** Farm account, farm management, fattening beef cattle, mobile app, user-centered

## Abstract

**Objective:**

This study aimed to develop a mobile application (app) specifically designed for enhancing farm management and performance assessment in fattening beef cattle.

**Materials and Methods:**

The development process followed a user-centered design approach, which involved focus group discussions and key informant interviews with 20 participants to design content and features. The app was developed for both mobile and web platforms. After the prototype and launch of the app, the system usability scale (SUS) and user satisfaction were assessed.

**Results:**

The assessment findings identify the specific expected functions in the app, with the farm accounting records function being the most desired feature among users, followed by production analysis, production records, and resource inventory. The mean SUS score was calculated to be 75.17, indicating a qualitative assessment of “Good.” The assessment of user satisfaction indicated that the mean satisfaction score for all participants was 4.26, suggesting a high level of satisfaction and a favorable perception of the app.

**Conclusion:**

This app provides an alternative way to record farm activity, suggest feed and feeding schedules, and provide financial management tools designed explicitly for small-scale beef cattle farmers.

## Introduction

The demand for meat protein is estimated to rise by 14% globally over the next decade, with an increase in beef production of 5.8% by 2030 compared to the years 2018–2020, mostly due to increasing income and population growth [1]. Thailand’s livestock production industry plays an important role in the agriculture economy, as it produces and exports poultry, swine, cattle, and aquaculture. The Ministry of Agriculture and Cooperatives estimated Thailand’s cattle sector’s gross domestic product at 1.2 trillion baht in 2021, or 37.2 billion United States dollar [2]. The Department of Livestock Development reported 9,394,111 cattle in Thailand in 2021, including 4,900,711 native cattle, 4,056,274 cross-bred cattle, 295,239 fattened cattle, and 141,887 pure-bred cattle [3]. Over the past five years, Thailand’s beef consumption has increased due to food service and tourism growth. Numerous supermarkets, hotels, restaurants, and online butcher shops sell medium-quality and premium beef [4]. However, the current demand for beef exceeds the available supply, leading to imports from countries such as Australia, New Zealand, Argentina, and the USA.

To meet this growing demand, beef cattle farmers must improve their productivity and efficiency while maintaining high animal welfare and environmental sustainability standards. A critical factor in achieving these objectives is effective farm management, which includes the monitoring and improvement of the productivity of each animal and the collective performance of the herd. Keeping records of livestock farm information provides numerous advantages, including enhanced management, improved health, increased efficiency, and better marketing. Many countries, especially the US, EU, Australia, Canada, and Brazil, restrict animal-derived product production and sales. Farmers can comply with rules and avoid legal issues by accurately recording livestock farm data. Traditionally, beef cattle farmers recorded data manually using paper systems. These methods may be time-consuming, make mistakes easily, and result in delayed data analysis.

Applications for mobile devices have grown rapidly along with smartphones, and agricultural apps have found numerous uses throughout the food production supply chain, especially in crop and livestock management. Livestock farmers could enhance efficiency, reduce costs, and increase profitability via a mobile app. Numerous mobile apps have been developed to facilitate daily tasks. A mobile application (app) for livestock could potentially incorporate various features, including livestock inventory management, health monitoring, feed management, breeding management, market information, and record-keeping [5–7].

Despite the development of mobile apps for keeping records of cattle operations on farms, there is still a significant knowledge gap regarding the lack of a mobile app specific to the effective monitoring of beef cattle during the fattening phase in Thailand. The significance of this knowledge deficit becomes especially apparent when considering the increasing beef market demand and the important economic contribution of the cattle industry to the country. The lack of a mobile app specifically designed for the management of beef cattle fattening presents challenges for small-scale beef cattle farmers, limiting their ability to improve operational efficiency, monitor livestock health, optimize feed management, and access market information suited to their requirements. The adoption of this technology offers significant opportunities for enhancing productivity, reducing production-related costs, and enhancing profitability in cattle production. Moreover, this coincides with the main objectives of Thailand 4.0, which emphasize the digitalization of various sectors and the advancement of technological developments.

In 2021, we launched the first version of the beef cattle management system, named Beef Cattle Farm Management (BCFM), for both Android and iPhone operating system (iOS) platforms. This system assists beef cattle farms with keeping their records, covering individual information, a vaccine and deworming program, treatment history, notifications, and so on. However, these apps lack essential features such as performance analysis, market information, and a farm account section, which could greatly contribute to more effective decision-making on the farm.

The objective of this study is to address the current knowledge gap by considering the implementation of a mobile app for smallholder beef cattle farmers and focusing on the management of fattening operations. The study focuses on various functionalities, including livestock inventory management, health monitoring, feed management, breeding management, market information, farm accounts, and record-keeping, using a user-centered design methodology that involves the active participation of all relevant stakeholders throughout the entire process. The adoption of a mobile app may provide advantages such as enhanced efficiency, reduced costs, and increased profitability, coinciding with the evolving demands of the cattle industry and the digital transformation goals of Thailand 4.0.

## Materials and Methods

### App development process

**Figure 1.**
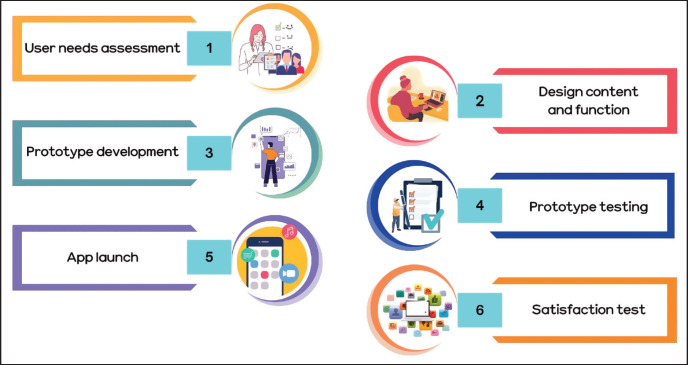
Process of user-centered design in app development.

The development process using a user-centered design approach consisted of six key phases: (1) assessment of user needs; (2) design of content and function; (3) development of prototypes; (4) testing of prototypes; (5) launch of the app; and (6) evaluation of user satisfaction. Figure 1 shows the user-centered design method used in the development of apps.

### User needs assessment

Focus group discussions and key informant interviews using semi-structured questionnaires fulfilled the functional requirements of the mobile app for smallholder beef-fattening cattle producers. Participating in focus group discussions and key informant interviews were 20 farms that use the BCFM app, which Saengwong et al. [8] developed in 2018. To understand the viewpoint and current circumstances experienced by smallholder farmers, we organized focus groups to present our conceptualization for the app. The questionnaire starts with basic demographic questions. The following section was a questionnaire regarding BCFM app usage. Finally, a survey was performed to collect participants’ needs and expectations for the app’s new features.

### Design content and function

During this phase, a group of researchers and developers employed data acquired from user needs assessments and a thorough review of previous research to create a wireframe for the app. This study identified appropriate content and functionality by considering the user’s requirements. In addition, we have analyzed the issues reported in the previous version of the app to guide the design of the current app.

### Prototype development

This system was developed on two platforms: a mobile app for the Android operating system and a web app. The app was created using the software tools Visual Studio Code and Android Studio and the structured query language database. Furthermore, the software exhibits a high degree of flexibility and has integrated capabilities with a wide variety of development environments.

**Figure 2.**
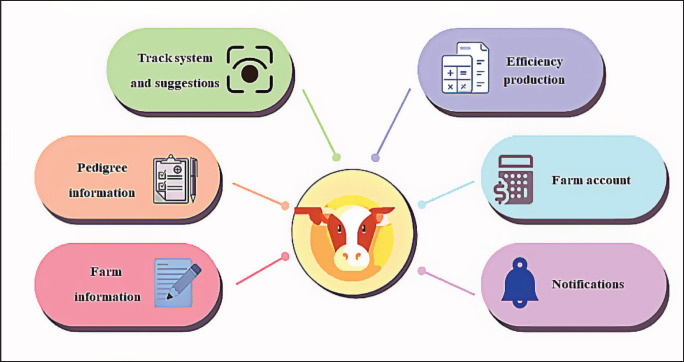
Conceptual framework of the app.

The latest update focuses on finishing beef cattle production through the enhancement of management recording functions in the BCFM app’s previous version. The feedlot, or finishing operation, is a stage in the beef industry’s production process related to the final feeding phase of cattle on a feedlot, which occurs just before the animals reach market weight. The primary aim of app design is to transform the conceptualization of an app into a functional and operational system. Figure 2 illustrates the conceptual framework utilized in developing the management app for fattening beef cattle farms.

### Prototype testing

Examination of usability before app launch was the primary goal of prototype testing. Twenty BCFM app-experienced smallholder beef cattle farmers participated in the assessment. The authors explained the objective that informed its development before presenting an overview of features and content. Following the installation of the app prototype, participants receive a checklist of tasks to perform, including testing the app, offering comments, and rating it. After collecting responses, the researchers assessed them systematically. The success rate was determined using the system usability scale (SUS) score, which comprised 10 standard questions with five response options and was measured with a five-point Likert scale with anchors for strongly agree and strongly disagree. Ethical approval to conduct the study was obtained from the University of Phayao Human Ethics Committee.

### App launch

After the app prototype has been tested successfully, the next stage in the development process involves the deployment of the app. The end-user can download the app from the Google Play store, which is developed for mobile devices that run on the Android operating system.

### Satisfaction test

The satisfaction test relates to the expressions of affection from reactions to a system, which are useful indicators of system quality characteristics. The evaluation process includes four aspects: aesthetics and design, usage, efficacy, and validity. The evaluation is performed using a five-point Likert scale, which includes responses from users indicating that they are very satisfied or very unsatisfied.

### Statistical analysis

The SUS score contains 10 items of mixed tone, with half of the questions (the odd numbers) having a positive statement and the other half (the even numbers) having a negative statement, all on a five-point Likert scale: 1 = strongly disagree, 2 = disagree, 3 = neutral, 4 = agree, and 5 = strongly agree. This calculation will provide a range of possible SUS scores from 0 to 100. The SUS is scored using the following formula:

SUS = ((Q1-1)+(Q3-1)+(Q5-1)-(Q7-1)+(Q9-1)+(5-Q2)+(5-Q4)+(5-Q6)+(5-Q8)+(5-Q10)) × 2.5

According to Bangor et al. [9], a SUS score of 52 is interpreted as “OK/fair” with low marginal acceptability ranges, a SUS score above 72 is considered acceptable with “good” usability levels, a SUS score above 85 corresponds to “excellent” usability levels, and a SUS score above 92 corresponds to “best imaginable” usability levels.

A five-point Likert scale was employed for evaluating user satisfaction: 5 indicated “extremely satisfied,” 4 “satisfied,” 3 “neutral,” 2 “unsatisfied,” and 1 “extremely unsatisfied.” The results of the average were transformed into means. A higher mean indicates a higher level of app preference. The interval for Likert scale satisfaction scores of app users was utilized (mean 1.00−1.80 = extremely unsatisfied, 1.81−2.60 = unsatisfied, 2.61−3.40 = neutral, 3.41−4.20 = satisfied, and 4.21−5.00 = extremely satisfied).

## Results

### User needs assessment

The desired content and function of the app were assessed using four multiple-choice questions. After discussion within the research group, we decided to design the questions in the questionnaire by separating them into four aspects: (1) content, (2) type of user interface, (3) format for showing data, and (4) specific function. The study involved selecting 20 participants based on an operating system that focuses on the rearing of fattened beef cattle as its primary objective. The participants were given the option to select multiple answers. Table 1 displays a list of questions from participants regarding the type of user interface of the app. Forages and pastures were the most desired content for consideration in the app, followed by feed and feeding, health, management, breeding and reproduction, market prices, and facilities. The evaluation results revealed that more than half of the participants preferred a graphical and colorful format, whereas the remaining participants favored clear text. Regarding the format for displaying data, 13 participants selected a graph, while the others chose a table. According to the fourth question, it was determined that the farm accounting records function was the most desired feature among users. Next, production analysis, production records, and resource inventory were all highlighted as desired features.

### Implementing contents and functions

**Figure 3.**
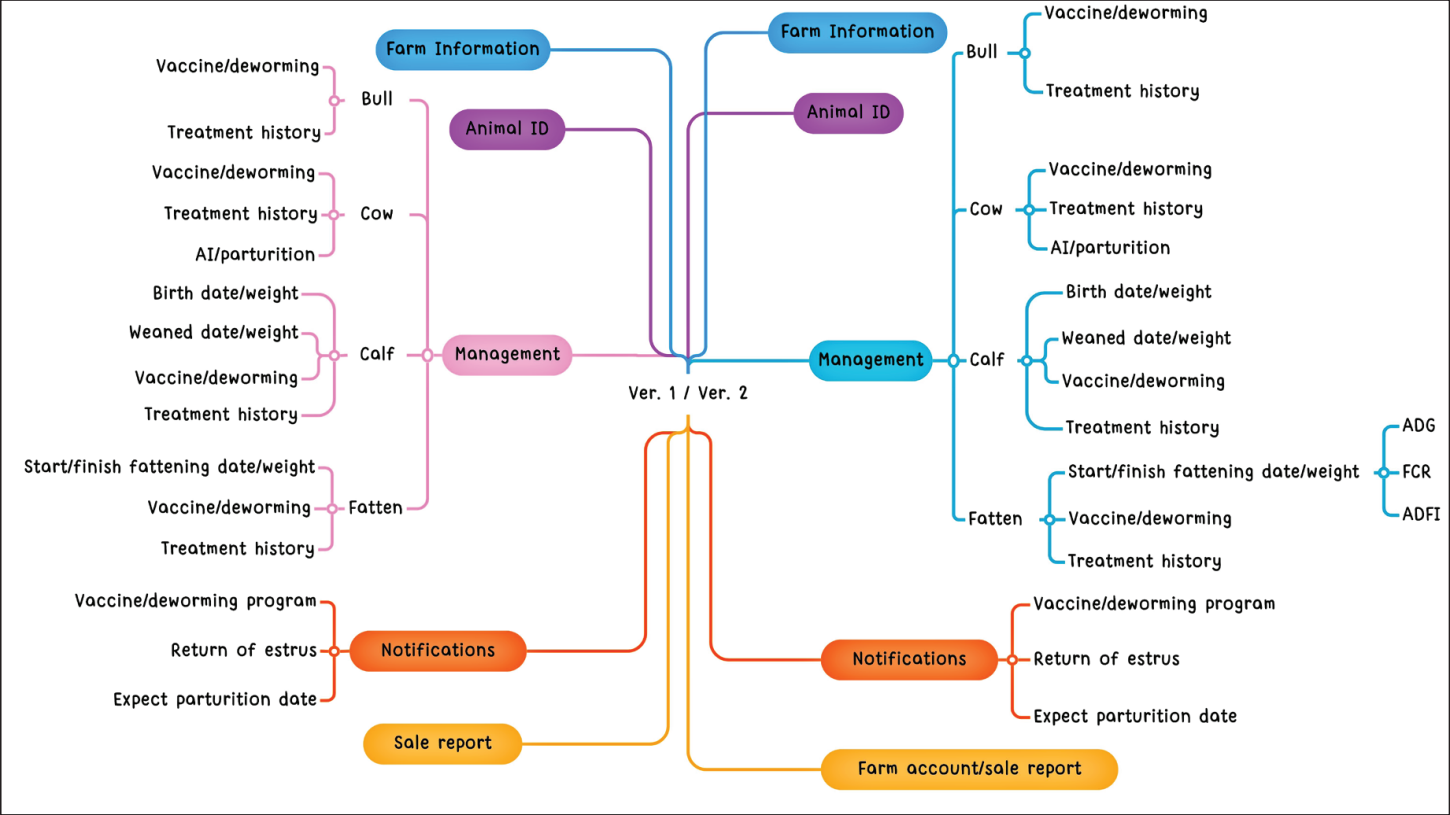
Mind map comparing the app’s current and previous versions’ features.

During this phase, all requested content and features that were identified via the user requirements assessment were effectively implemented. The app considered all feedback regarding the format, user interface, function, and content. Figure 3 illustrates the extracted functions and how they relate to the objectives of mobile apps and are compared with the previous version.

**Figure 4.**
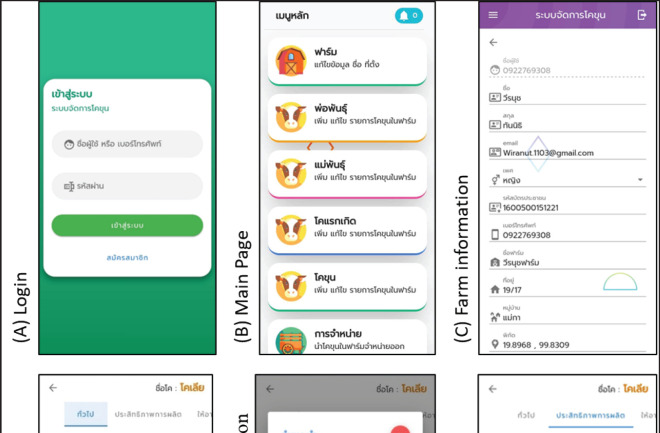
(A)–(I) Screenshots of the user interface design of the app.

Figure 4(A-I) shows the main app screenshots. The login interface is shown in the first screenshots of Figure 4(A). New users have to respond to questions to register on the app. This includes the user’s full name, gender, age, username, and password. After responding to the questions and entering their username and password, users could click “log in.” Similar to other apps, users are able to ask for support online if they forget their account and password. The nine-menu main page user interface is shown in Figure 4(B). The menus are: (1) farm information; (2) bull or sire information; (3) cow information; (4) calf information; (5) fattening information; (6) sale report; (7) farm account; (8) knowledge dissemination; and 9) farm report. These menus feature the interface’s main functions. In Figure 4(C), farm information, a new user would fulfill the farm location. The farm’s position was determined by the global positioning system (GPS). Figure 4(D) helps users identify bulls, cows, calves, and fattens by production stage. Figure 4(E) shows fattening feed recommendations. This menu suggests feed ingredient composition and feeding amounts for fattening. Figure 4(F) measures animal efficiency. This function assists farmers in assessing farm productivity using ADG, feed conversion ratio, ADFI, and so on. Figure 4(G) shows vaccine and deworming notification. The user gets a reminder of the vaccinations and deworming program. Figure 4(H) displays a farm accounting record, one of the farmer’s most important functions for tracking income and expenses. This will help farmers make better investment and spending decisions and manage their finances. The last screenshot in Figure 4(I) shows knowledge dissemination content. The topic at issue relates to strategies for optimizing cattle weight gain to maximize financial returns. Overall, with the proper use of mobile apps in agriculture, farmers can improve their productivity and profitability by gaining quick access to critical information.

**Table 1.** The list of questions in the user needs assessment questionnaire and a summary of user expectations on the app’s functions and content (*n* = 20).

Contents and features	Value (*n*)
1. What content would you like to feature in the app? - Breeding and reproduction - Feed and feeding (nutrition) - Management - Health (disease and treatment) - Facilities - Forages and pastures - Market prices	8 12 9 10 5 15 7
2. What type of app’s user interface works for you the best? - Clean text - Graphical and colorful	4 16
3. What is the preferred format for displaying data when using the app? - Table - Graph	7 13
4. What specific functions are expected to be included in the app? - Farm accounting records - Production records - Resource inventory - Production analysis	14 11 8 12

### Usability and satisfaction tests

Table 2 presents the mean and standard deviations of the raw scores for each question. The principal value of the SUS score is that it provides a single reference score for participants’ views of a system’s usability. The average value of the first to tenth questions in the questionnaire was calculated at 75.17, resulting in a qualitative rating of “good” for the SUS. This means the user liked the app, found it user-friendly, and felt confident that they would be able to learn how to use it quickly and without technical assistance.

Participants were required to fill out a survey to collect information and assess the level of user satisfaction. The primary objective was to assess the user interface by using a five-point Likert scale divided into four-part categories: aesthetics and design, usage, efficiency, and validity of the app. The greater the overall score, the more satisfied the users were. The mean and standard deviation for each measurement are briefly presented in Table 3. The results of the user satisfaction evaluation indicated that a mean score of 4.26 was received by all participants, demonstrating that they were extremely satisfied with the app and had a positive opinion about it. The study findings indicated that participants expressed satisfaction with the app’s validity, aesthetics and design, usage, and efficacy, with respective scores of 4.22, 4.28, 4.19, and 4.33.

**Table 2.** Responses to the app’s individual SUS score (*n* = 30).

SUS questions	Mean (SD)	Range
I think I should use the app frequently.	3.97(0.72)	3–5
I found the app quite complex.	1.70(0.65)	1–3
I found the app user-friendly.	4.00(0.69)	3–5
It seems that I would require technical assistance while using the app.	1.70(0.84)	1–3
The app’s functions were well integrated.	4.03(0.72)	3–5
The app lacks consistency.	1.70(0.60)	1–3
Most users seem to have no trouble learning the app.	4.03(0.61)	3–5
I found the app difficult to use.	2.10(0.80)	1**–**3
I felt confident using the app.	3.53(0.63)	3**–**5
I had to learn a lot before using this app.	2.30(0.65)	1–3
**Calculated average SUS score**	**75.17(7.10)**	

1 = strongly disagree, 2 = disagree, 3 = neutral, 4 = agree, and 5 = strongly agree.

**Table 3.** Summary of user satisfaction (*n* = 30).

Criterion category	Evaluation		
	x̄	SD	Level
1. The aesthetics and design	4.22	0.80	Extremly satisfied
2. The usage of the app	4.29	0.81	Extremly satisfied
3. The efficiency of the app	4.19	0.80	Satisfied
4. The validity of the app	4.33	0.71	Extremly satisfied
**Overall average mean**	4.26	0.78	Extremly satisfied

5 = extremly satisfied, 4 = satisfied, 3 = neutral, 2 = unsatisfied, and 1 = extremly unsatisfied

## Discussion

Developing a livestock management mobile app provides several benefits: 1) improved efficiency: mobile apps enable farmers to track and monitor their livestock online at any time, increasing farm efficiency. This can improve time management and reduce manual work [10–12]. 2) Better data management: mobile apps offer farmers real-time data about their livestock’s health, feeding conditions, and growth rates. This information could help farmers make better herd management and breeding decisions [13,14]. 3) Improve animal health: Farmers may quickly notify health problems and take preventative actions via real-time data on their livestock. Prevention could reduce disease spread and use of antibiotics [15], and 4) increase customer trust: mobile apps will assist farmers in providing consumers with detailed information about their products, including raising conditions. Increase profits and customer loyalty by promoting trust and satisfaction. Global livestock farm record-keeping mobile apps enable users to manage their farm information. These apps provide farmers with records of breeding history, health care, feed regimen, sale, and marketing data for their cattle [16–18].

The development of this app aimed to provide smallholder beef cattle farmers with an alternative method for record-keeping to reduce time requirements and enhance farm productivity. The goal of this study was to thoroughly describe the user-centered design process used in the development, creation, and implementation of this app for Thai smallholder beef cattle farmers. This design not only implements a process approach to design in which developers consider the desires and needs of users at all stages, but it also offers the most efficient results and a suitable framework for app development. The app’s features enable users to record animal activity on the farm with notifications, suggest feed and feeding schedules, and assist farmers with financial tracking using financial management tools. In addition, these apps disseminate knowledge about raising beef cattle, breeding techniques, nutrition management, pasture management, and other relevant topics. This study represents the first investigation conducted in Thailand concerning the adoption of mobile apps for recording systems on beef cattle fattening farms. Based on the present study, it has been found that the user expressed a high level of satisfaction with the aesthetics, design, usage, and validity of the app. In addition, adopting the tool may provide significant benefits in terms of efficiency, data management, productivity, animal health, and customer confidence.

The study’s strengths were demonstrated through the active involvement of end users throughout the app’s design, development, and implementation phases. The app has the potential to assist the user in addressing issues with farming by optimizing farm efficiency and decreasing working time. First, it enhances efficiency by providing valuable data to help producers make accurate choices about feeding schedules and nutrient recommendations. Second, it alerts farmers to vaccine and deworming schedules. Third, it reduces costs by assisting farmers in managing their farms more efficiently, thereby enabling them to save significantly on expenses. Fourth, it educates farmers on management practices that optimize beef cattle production. Finally, the app’s accessibility is essential, as it can be accessed anywhere and anytime.

There are several limitations that necessitate careful consideration regarding this project. One limitation that was related to the development of this app was the Android operating system. Additional research is necessary related to the conception, creation, and implementation of the app on the iOS platform or alternative operating systems. The limited area of functionality may not be able to provide all the requirements for efficient livestock management. Depending on internet connectivity problems, the app may not be inaccessible or may not function properly. Not all livestock owners or users may be familiar with using a mobile app or have access to a smartphone, and they may not be concerned with the efficiency of data utilization. Smallholder farmers cannot afford the high maintenance costs of mobile apps and have to rely on subsidies from the government. The current matter refers to security concerns, where specific mobile apps with confidential data, such as livestock health records, may be at risk of cyber-attacks or breaches of data. The potential consequences of affecting the privacy and security of information extend to the well-being of both animals and their owners.

## Conclusion

The profitability and efficiency of livestock farms are dependent on a variety of aspects, including genetics and breeding, feeding strategies, market demand, and pricing dynamics, in addition to the implementation of effective management practices. Effective record keeping is an essential element that impacts the implementation of good management practices aimed at increasing farm productivity. The recording of farm activities covers the identification of animals, their feeding schedule, health status, treatment methods, and reminder notifications, and provides useful information. The “Khokun” Android app is mobile software that offers a user-friendly interface designed to help smallholder farmers enhance their farm production. Future research utilizing the app methodology could implement these steps and learn from this experience to better design solutions to increase beef cattle farm production.

## List of Abbreviations

ADG, average daily gain; ADFI, average daily feed intake; App, application; BCFM, Beef Cattle Farm Management; iOS, iPhone operating system; SUS, system usability scale.

## References

[1] (2023). FAO. Meat.

[2] MOAC (2023). Gross Domestic Product: Q4/2022.

[3] DLD (2023). Number of livestock inventory in Thailand.

[4] USDA (2023). Thailand’s Beef Market.

[5] Do QD, Tran TT, Truong TB, Do NT, Hoang LK (2023). Determinants of smartphone adoption and its benefits to the financial performance of agricultural households: evidence from Hoa Binh province. Vietnam. Asian J Agric Dev.

[6] Gao Y, Zhao D, Yu L, Yang H (2020). Influence of a new agricultural technology extension mode on farmers’ technology adoption behavior in China. J Rural Stud.

[7] Kakani VP, Debasis G, Arunasis G, Chanchal D (2021). Animal husbandry mobile apps in transformation livestock farming. Int J Curr Microbiol App Sci.

[8] Saengwong S, Thannithi W, Intawicha P, Porkaew C (2021). Development of a mobile app for recording and management alert on-farm to supporting beef cattle smallholder farmers. Int J Agric Technol.

[9] Bangor A, Kortum PT, Miller JT (2008). An empirical evaluation of the system usability scale. Intl J Hum-Comput Interact.

[10] Liu C, Jian Z, Xie M, Cheng I (2021). A real-time mobile application for cattle tracking using video captured from a drone. In 2021 International Symposium on Networks, Computers and Communications (ISNCC).

[11] Barriuso AL, Villarrubia GG, De Paz JF, Lozano Á, Bajo J (2018). Combination of multi-agent systems and wireless sensor networks for the monitoring of cattle. Sensors.

[12] Vannieuwenborg F, Verbrugge S, Colle D (2017). Designing and evaluating a smart cow monitoring system from a techno-economic perspective. Internet of Things Business Models, Users, and Networks.

[13] Kabir MA, Sultana N, Al Noman AA, Jahangir Hossain SJ, Miraz MFH, Deb GK (2022). FeedMaster: a least-cost feed formulation app for minimizing the cost and maximizing milk yield. J Adv Vet Anim Res.

[14] Herrera K, Miranda J, Mauricio D (2022). Milchbot: app to support the process of feeding and caring for dairy cows in Peru. Agris On-line Pap Econ Inform.

[15] Shanka D, Genale A (2022). Mobile application based expert system for cattle disease diagnosis and treatment in Afan Oromo language. Int J Informatics Inf Syst.

[16] Fouad K, Alary V, Dubron A, Bonnet P, Juanes X, Nigm A (2021). Developing a data collection application for following up the small-scale dairy farms’ performance in rural areas. Egypt J Anim Prod.

[17] Darvesh K, Khande N, Avhad S, Khemchandani M (2023). IOT and AI based smart cattle health monitoring. J Anim Sci.

[18] Louta M, Karagiannis P, Papanikolopoulou V, Vouraki S, Tsipis E, Priskas S (2023). FarmDain, a decision support system for dairy sheep and goat production. Animals.

